# Satisfaction with medication in older adult patients with chronic respiratory diseases: a multicenter cross-sectional observational study

**DOI:** 10.3389/fpubh.2023.1168249

**Published:** 2023-08-21

**Authors:** Jiankang Wu, Weiwei Meng, Huihui Zeng, Yiming Ma, Yan Chen

**Affiliations:** Department of Pulmonary and Critical Care Medicine, The Second Xiangya Hospital, Central South University, Changsha, Hunan, China

**Keywords:** older adult patients, chronic respiratory diseases, COPD, TSQM-II, related factors

## Abstract

**Purpose:**

To gain insight into medication satisfaction and factors associated with chronic respiratory disease, particularly chronic obstructive pulmonary disease (COPD) in older adults, focusing on public health issues and improving the health of the older adult population.

**Methods:**

This cross-sectional study was conducted from October 2022 to November 2022 in 24 hospitals in different regions of Hunan Province, China. Older adult patient treatment satisfaction was assessed using the Treatment Satisfaction Questionnaire for Medication version II. Multiple regression analysis was used to identify factors independently associated with patient treatment satisfaction.

**Results:**

Only 15.9% of all patients scored above 80 in the effectiveness domain, while 11.6 and 16.5% scored above 80 in the convenience and global satisfaction domains, respectively, while 17.3% reported having side effects. Interstitial lung disease was associated with lower drug satisfaction than other disorders (*p* < 0.05). Multifactorial regression analysis showed that age, education background, profession, and smoking status were independently associated with satisfaction among patients with chronic respiratory diseases (*p* < 0.05). Education background, profession, CAT score, number of acute exacerbations, duration of home oxygenation and duration of home ventilator use were independently associated with satisfaction in patients with COPD (*p* < 0.05).

**Conclusion:**

Low satisfaction with chronic respiratory drug therapy was associated with age, education background, profession and smoking status. Satisfaction was lower for patients with interstitial lung disease. For COPD, CAT score, education background, profession, number of acute exacerbations, home oxygen and ventilator use influence satisfaction. Clinicians can identify appropriate patients and communicate effectively with them throughout treatment and follow-up, vigorously promote smoking cessation and home oxygen therapy, increase medication satisfaction, especially among older adults, and in turn improve public health and the quality of life of older adults.

## 1. Introduction

Chronic respiratory diseases are diseases of the respiratory tract and other structures of the lung and are among the leading causes of morbidity and mortality worldwide (1, 2). The most common chronic respiratory diseases are asthma, chronic obstructive pulmonary disease (COPD), and occupational lung diseases such as pneumoconiosis, thus contributing to the global burden of noncommunicable diseases with negative social and economic consequences (3). Although there is no cure for chronic respiratory disease, various forms of treatment can help control symptoms, improve patients’ quality of life, and prevent the adverse outcomes (including exacerbation) associated with increased morbidity, increased health care use, disability, and risk of death (4). Statistics also reveal that the financial impact of treating chronic respiratory disorders on the economy of all nations is rising. Care for individuals with chronic respiratory disorders alone costs roughly €380 billion annually in the 28 EU member states as of 2019 (5). Importantly, chronic respiratory diseases disproportionately affect older adults, who often experience age-related physiological changes and are more susceptible to respiratory impairments (6). The aging population poses unique challenges in the realm of public health, as it necessitates a comprehensive understanding of the specific needs and considerations of older adult individuals (7). Aging is characterized by a complex interplay of biological, psychological, and social factors that can influence medication satisfaction and treatment outcomes (8). Therefore, public health efforts aimed at addressing chronic respiratory diseases must consider the implications of aging both from a population health standpoint and when working directly with older adult patients.

Commonly used drugs for chronic respiratory diseases are cough suppressants, expectorants, asthma suppressants and glucocorticoids. Cough suppressants mainly include central and peripheral cough suppressants. Expectorants are mainly divided into nausea expectorants, stimulant expectorants and mucolytics. Asthma medications are divided into bronchodilators, anti-allergic asthma medications and anti-inflammatory asthma medications. Glucocorticoids include inhaled glucocorticoids and oral glucocorticoids. If the respiratory tract infection is combined with bacterial infection, various antibiotics are also required for treatment (9–13). For example, COPD and asthma, are among the most common chronic respiratory diseases for which licensed therapies are effective in reducing symptom burden, improving health-related quality of life, and maintaining or slowing disease progression. However, reports of a large number of asthma and COPD exacerbations and associated stressors to emergency and respiratory care persist (14). The reason for this is mainly that efficacy outcomes from randomized controlled trials are usually characterized as well-controlled, highly selective, and short-term, whereas in real-life efficacy assessments often involve different patient populations, different care settings and patient characteristics, and longer time intervals (15). A study involving 401 people assessed the satisfaction of Italian COPD patients with their medication use. The study found that COPD patients were only moderately satisfied with their treatment. High patient satisfaction was mainly associated with low awareness of the disease, high adherence to treatment and lower levels of pain (16). Real-world data is essential to assess the effectiveness of licensed therapies, given the differences in the patient population and settings in which they are used. Indeed concerns regarding drug efficacy, adverse effects, dosing regimen and length of treatment are worthwhile. Both subjective and objective feelings can have a significant impact on a patient’s adherence to the medication, which is crucial for the effective management of the disease. For example, if a patient experiences unpleasant side effects or discomfort while taking the medication, they may be less likely to adhere to the prescribed dosage or even stop taking the medication altogether. In contrast, if a patient perceives that the medication is working well and improving their symptoms, they may be more likely to stick to the treatment plan and achieve better outcomes.

The Treatment Satisfaction Questionnaire for Medication II (TSQM-II) is a validated instrument for investigating patient satisfaction with medication (17). The TSQM-II has been validated in a heterogeneous population and the questionnaire has been shown to have high internal consistency (18). In addition, the questionnaire has been used to assess medication satisfaction in a variety of conditions (19–21), and have proved that the Chinese version of the TSQM-II also has been validated in the Chinese population (22). Supplementary Table 2 provides an overview of the application of TSQM II in China and other countries. Therefore, exploring medication satisfaction among older adult individuals with chronic respiratory diseases within the broader public health context is essential for optimizing their care and well-being. The aim of this study was to investigate medication satisfaction in older adult patients with chronic respiratory diseases, especially the most common COPD patients, and to explore potential associated factors in China. The findings may offer insights and evidence to enhance patient medication satisfaction and inform shared decision-making in clinical practice.

## 2. Materials and methods

### 2.1. Patients

**Figure 1:**
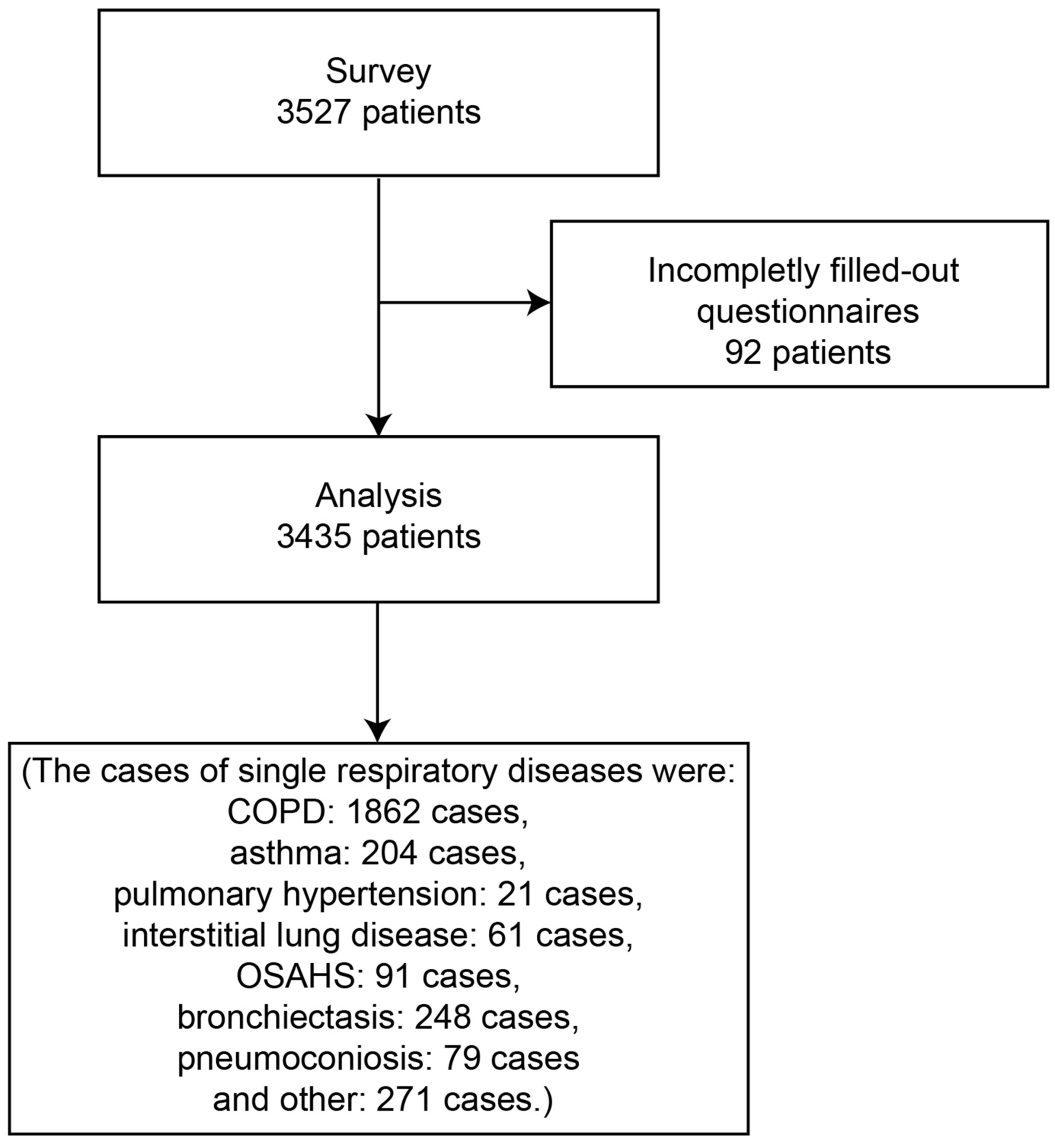
Patient flowchart.

3,527 patients were recruited between October 2022 and November 2022 in 24 hospitals in different cities, counties and districts in Hunan Province, China, and information was collected using a self-administered questionnaire. Inclusion criteria were a history of ≥1 of the following previous medical conditions: respiratory diseases such as COPD, asthma, pulmonary hypertension, interstitial lung disease, obstructive sleep apnea/hypopnea syndrome (OSAHS), bronchiectasis, pneumoconiosis and other. Figure 1 is the flow chart of this study.

### 2.2. Methods

The general information questionnaire was developed by the investigators themselves after reviewing the literature and integrating expert opinions, and collected baseline information on age, gender, education background, profession, location developed/underdeveloped, smoking status, and residence (urban/rural). Among them, CAT score, mMRC score, home oxygen therapy, home non-invasive ventilation, and baseline information on history of acute exacerbation of COPD in the previous year were also collected for patients who filled in that they had COPD, which was defined as worsening symptoms requiring antibiotic medication, systemic corticosteroids (moderate), hospitalization, or a combination of the above (severe). The main observation was the second version of the satisfaction with medication (TSQM), which contains 11 questions and can be divided into four sections: effectiveness score, side effect score, convenience score, and global satisfaction score, with a score range between 0 and 100, with higher scores associated with higher satisfaction levels. TSQM-II was previously used in China and the scores were calculated in the same way as in the original paper by Atkinson et al. (18, 23). Effectiveness: ([(Item 1 + Item 2) − 2] divided by 12) × 100. Side effects: ([(Sum of Item 4 to Item 6) − 3] divided by 12) × 100. Convenience: ([(Sum of Item 7 to Item 9) − 3] divided by 18) × 100. Global satisfaction: ([(Sum of Item 10 to Item 11) − 2] divided by 12) × 100. The study used voluntary responses to collect the questionnaires, and the questionnaires with complete information and no logical errors in the answers to the questions were judged as valid responses, otherwise they were not qualified. After collecting all the answer sheets, the proportion of each option for each question was counted, and the answer data were compared and analyzed.

### 2.3. Statistical analysis

SPSS 26.0 (IBM, NY, United States) was used for statistical analysis. The continuous variables were tested for normal distribution using the Kolmogorov–Smirnov test. The mean ± standard deviation (SD; normal distribution) and median (range; skewed distribution) were calculated for continuous variables. The Mann–Whitney *U*-test (skewed distribution) and the Student’s *t*-test (normal distribution) were used for analysis. Categorical variables are presented as frequency (percentage) and were analyzed using the Chi-square test. The univariable linear regression analyses were performed for baseline characteristics of the patients to identify factors associated with the global satisfaction of patients (*p* < 0.05). Then, the significant variables were entered into a multivariable linear regression. The linear regressions were performed using the standardized TSQM-II total scores. *p*-values < 0.05 were considered statistically significant.

## 3. Results

### 3.1. Characteristics of the study population

**Figure 2:**
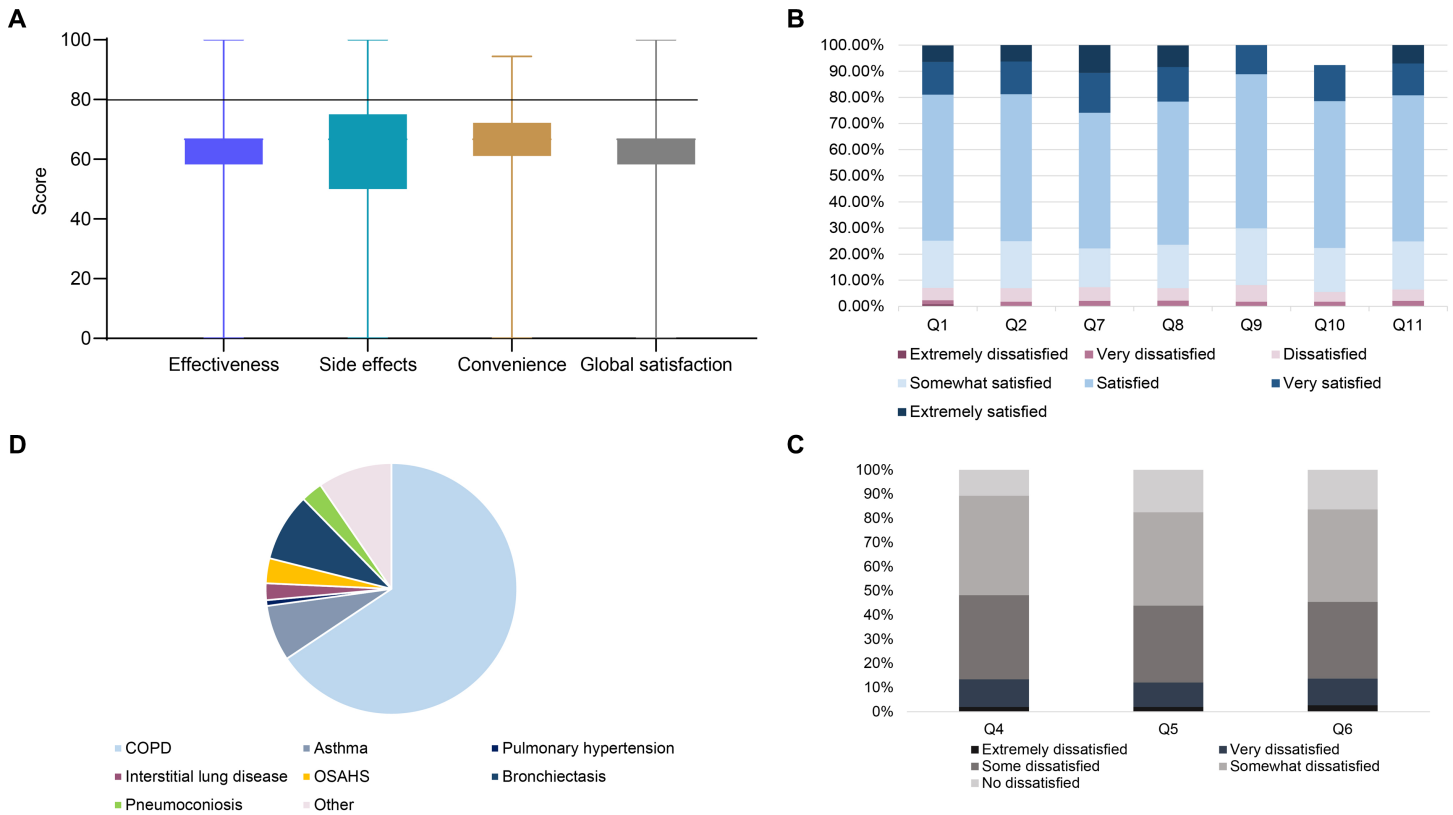
**(A)** Boxplots for treatment satisfaction evaluated by the TSQM-II. **(B)** Stacked bar chart distribution of responses of patients with chronic respiratory disease to TSQM-II questionnaire items in the domains of effectiveness (Q1–Q2), convenience (Q7–Q9) and global satisfaction (Q10–Q11), with answers ranging from extremely dissatisfied (dark pink) to extremely satisfied (blue) on a scale of 7. Q1 indicates “ability to prevent or treat,” Q2 indicates “relief of symptoms,” Q7 indicates “ease of medication,” Q8 indicates “ease of when to take medication,” Q9 indicates “frequency of medication/medication”; Q10 indicates “more benefits than disadvantages of medication,” and Q11 indicates “satisfaction considering everything.” **(C)** The stacked bar chart shows the distribution of the responses of patients with chronic respiratory diseases to the side effect domains of the TSQM-II questionnaire on a 5-point scale from extremely dissatisfied (black) to not dissatisfied (gray). Q5 means “side effects of thinking ability (e.g., ability to think clearly, stay awake)”; Q6 means “side effects of emotions or moods (e.g., anxiety, fear, sadness, irritation).” **(D)** The pie chart shows the distribution of patients with a single chronic respiratory disease condition.

A total of 3,527 questionnaires were sent out in this study, 92 were not completed after answering or failed the questionnaire, and 3,435 valid questionnaires were recovered, the recovery rate was 97.4%. Baseline information of patients with chronic respiratory diseases and their medication satisfaction scores are shown in Table 1. The patients were 67.10 ± 13.13 years old and 69.1% were male. The proportion of patients who are smoking is 18.1%, and the proportion of patients who have smoked before and never smoked is 41.4% and 40.6%, respectively. Further statistics yielded single respiratory diseases as COPD: 1,862 cases, asthma: 204 cases, pulmonary hypertension (including chronic pulmonary heart disease): 21 cases, interstitial lung disease: 61 cases, OSAHS: 91 cases, bronchiectasis: 248 cases, pneumoconiosis: 79 cases and other: 271 cases, respectively (Figure 2D). Baseline information about our patients with COPD of special interest is shown in Supplementary Table 1. In total, there were 2,218 COPD patients, 71.8% of whom were males, thus 356 patients with other combined respiratory diseases.

**Table 1:** Characteristics of the patients.

Characteristics	Total (*N* = 3,435)
Age (years), mean (SD)	67.10 (13.13)
**Sex, *n* (%)**	
Male	2,375 (69.1)
Female	1,060 (30.9)
**Education background, *n* (%)**	
Primary school and below	1,585 (46.1)
Junior high school	1,052 (30.6)
High school or technical or vocational school	528 (15.4)
Bachelor degree or above	270 (7.9)
**Local area, *n* (%)**	
Developed area	2,506 (73.0)
Less developed area	929 (27.0)
**Profession, *n* (%)**	
Agriculture, forestry, animal husbandry, fishing, water production personnel	1,400 (40.8)
Professional and technical personnel	204 (5.9)
Production and transportation equipment operators and related personnel	161 (4.7)
Commercial service personnel	144 (4.2)
State organs, Party and mass organizations, enterprises, institutions;	280 (8.2)
Medical and health related personnel;	103 (3.0)
Others	1,143 (33.2)
**Place of residence, *n* (%)**	
Urban area	1,318 (38.4)
Rural area	2,117 (61.6)
**Smoking status, *n* (%)**	
Be smoking	620 (18.0)
Previous smoking	1,420 (41.4)
Never smoked	1,395 (40.6)
**Patients with a single respiratory disease**	
COPD	1,862
Asthma	204
Pulmonary arterial hypertension	21
Interstitial lung disease	61
Sleep apnea syndrome	91
Branch expansion	248
Pneumoconiosis	79
Other	271

### 3.2. Scores of satisfaction

In Figure 2, patient satisfaction with medications for chronic respiratory disease, as measured by the TSQM II, was low, with only 15.9% of patients scoring above 80 in the effectiveness domain, compared with 11.6% and 16.5% in the convenience and overall satisfaction domains, respectively. The median total satisfaction score (25th–75th percentile) of 66.7 (58.3–66.7) in the effectiveness domain, with 74.8% of patients satisfied with the ability of the medication to prevent or treat, and 75.1% satisfied with the relief of symptoms caused by the medication; in the convenience domain median satisfaction was 66.7 (61.1–72.2), 77.8% of patients were satisfied with the ease of using the medication, 76.30% were satisfied with the ease of planning when to use the medication, and 70.10% were satisfied with the frequency of the medication; in the global satisfaction domain median satisfaction was 66.7 (58.3–66.7), 70.0% of patients were satisfied with the extent to which the benefits of the medication outweighed the disadvantages, and 75.7% of patients were satisfied with the medication when all things were taken into account. In addition patients reported side effects at a rate of 17.3% with a median overall score (25th–75th percentile) of 66.7 (50.0–66.7), with 45.4% of patients dissatisfied with side effects that interfered with physical health and ability to work (e.g., strength, energy), 48.2% dissatisfied with side effects that interfered with the ability to think (e.g., ability to think clearly, stay awake), and 43.9% of the patients were dissatisfied with the side effects that affected their emotions or mood (e.g., anxiety, fear, sadness, irritation).

**Figure 3:**
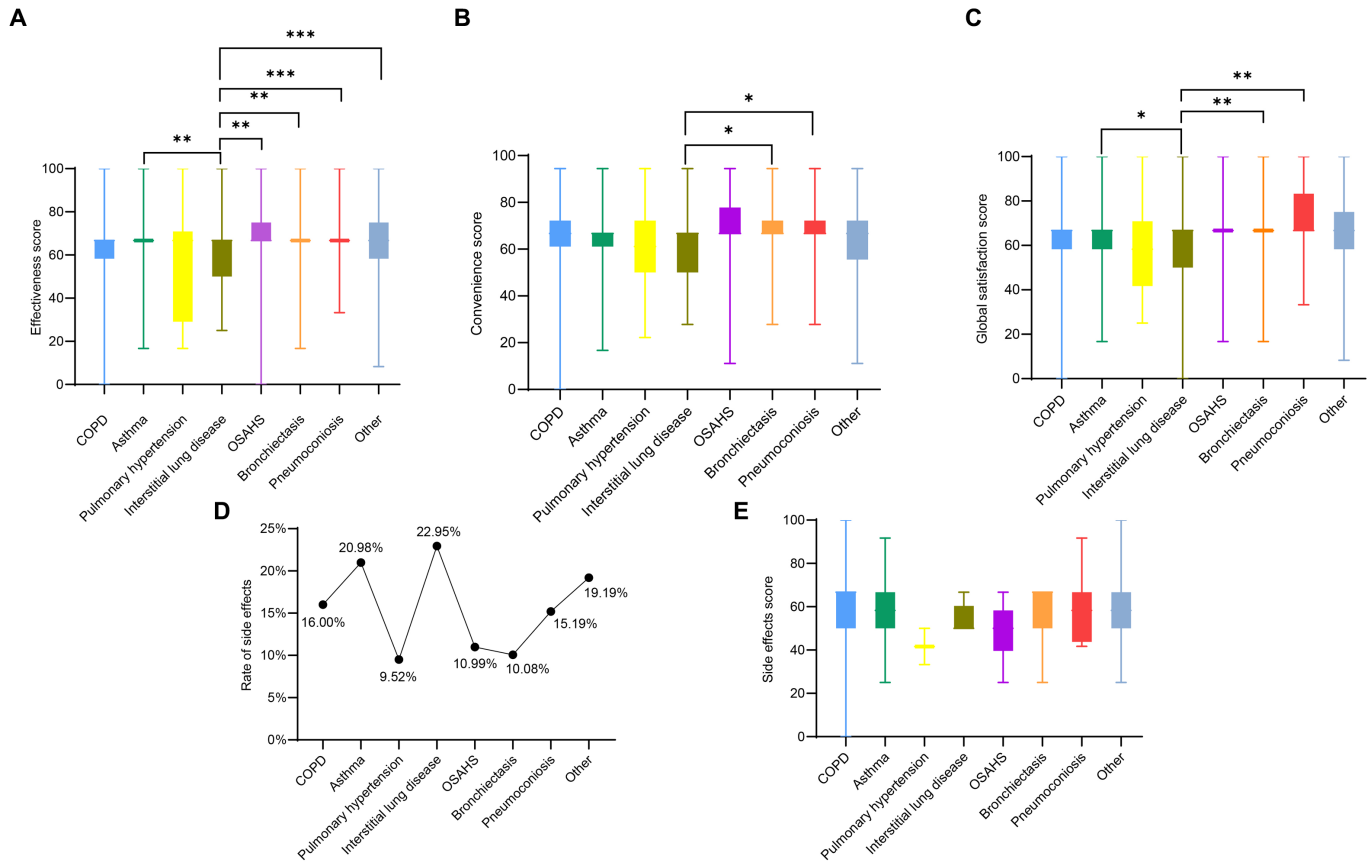
Panels **(A–E)** are the effectiveness domain score, convenience domain score, global satisfaction domain score, side effect rate, and side effect domain score for patients with a single chronic respiratory disease, respectively. *p < 0.05, **p < 0.01, ***p < 0.001.

In Figure 3, the distribution and characteristics of TSQM-II scores for single chronic respiratory diseases, including COPD, asthma, pulmonary hypertension, interstitial lung disease, OSAHS, bronchiectasis, pneumoconiosis, and other, are shown. It can be concluded that interstitial lung disease has lower effectiveness, convenience and global satisfaction scores than other diseases. In addition Figures 3D,E demonstrate the side effect reporting rates and side effect scores for different diseases, with interstitial lung disease being the highest.

### 3.3. Factors influencing satisfaction

Table 2 presents the results of multivariate linear regression used to determine the factors influencing patient satisfaction with medications. The results showed that in the effectiveness domain, patient satisfaction was positively associated with junior high school (*p* = 0.001), high school/junior high school/vocational school (*p* = 0.009), and university college/bachelor’s degree and above (*p* = 0.007), patient satisfaction was also positively associated with personnel in state agencies, party organizations, enterprises, and institutions (*p* = 0.024) and health care-related practitioners (*p* < 0.0001) occupation was positively correlated, and patient satisfaction was positively correlated with never having smoked (*p* = 0.001). In the side effects domain, age was positively associated with patient satisfaction (*p* = 0.006) and patient satisfaction was positively associated with junior high school education (*p* < 0.0001). In the convenience domain, junior high school education (*p* = 0.002) and never having smoked (*p* = 0.001) were positively correlated with patient satisfaction, in addition, patient satisfaction was also positively correlated with the occupation of personnel in state agencies, party organizations, enterprises, and institutions (*p* = 0.036) and health care-related practitioners (*p* < 0.0001). In the area of global satisfaction, patient satisfaction was positively correlated with junior high school education (*p* < 0.0009), patient satisfaction was also positively correlated with personnel in state agencies, party organizations, enterprises, and institutions (*p* = 0.005) and health care-related practitioners (*p* < 0.0001) occupation, and finally previous smoking (*p* = 0.04) and never smoking (*p* = 0.001) were positively correlated with patient satisfaction was positively correlated.

**Table 2:** Factors influencing medication satisfaction, based on TSQM.

	Effectiveness		Side effect		Convenience		Global satisfaction	
	Coefficient	*P-*value	Coefficient	*P-*value	Coefficient	*P-*value	Coefficient	*P-*value
Age (years)	−0.014	0.548	0.202	0.006	−0.028	0.204	−0.036	0.117
Male (Ref. female)	--	--	--	--	--	--	−0.005	0.995
**Education background (Ref. primary school and below)**								
Junior high school	2.209	0.001	7.373	<0.0001	1.878	0.002	1.638	0.009
High school or technical or vocational school	1.118	0.009	2.129	0.083	0.669	0.092	0.467	0.260
Bachelor degree or above	1.186	0.007	−0.326	0.784	0.702	0.085	0.349	0.413
**Local Area (Ref. less developed area)**								
Developed area	0.756	0.215	--	--	−0.729	0.197	--	--
**Profession (Ref. agriculture, forestry, animal husbandry, fishing, water production personnel)**								
A	−0.728	0.548	--	--	1.229	0.274	1.601	0.174
B	−0.559	0.391	--	--	0.159	0.793	0.086	0.892
C	0.283	0.303	--	--	0.438	0.085	0.290	0.279
D	0.806	0.024	--	--	0.695	0.036	0.983	0.005
E	0.415	<0.0001	--	--	0.387	<0.0001	0.405	<0.0001
F	−0.728	0.548	--	--	1.229	0.274	1.601	0.174
**Smoking status (Ref. be smoking)**								
Previous smoking	1.488	0.050	--	--	1.233	0.079	1.514	0.040
Never smoked	1.257	0.001	--	--	1.153	0.001	1.504	0.001

A, Professional and technical personnel; B, Production and transportation equipment operators and related personnel; C, Commercial service personnel; D, State organs, Party and mass organizations, enterprises, institutions; E, Medical and health related personnel; F, Others.

Table 3 presents the results of multivariate linear regression used to determine the factors influencing COPD patients’ satisfaction with their medications. The results showed that in the effectiveness domain, patient satisfaction was positively associated with junior high school (*p* = 0.011) and high school/secondary/vocational school (*p* = 0.001) education, patient satisfaction was also positively associated with other occupations (*p* = 0.003), patient satisfaction was negatively associated with CAT score (*p* < 0.0001), and patient satisfaction was positively associated with acute exacerbations 1–3 times (*p* = 0.002), furthermore, patient satisfaction was positively correlated with home oxygen therapy time (*p* < 0.005), however, patient satisfaction was negatively correlated with home non-invasive ventilation time (*p* < 0.005). In the area of side effects, side effects were reported by 17.5% of COPD patients, patient satisfaction was positively associated with mMRC score (*p* < 0.0001). But patient satisfaction was negatively associated with CAT score (*p* < 0.0001) and home non-invasive ventilation time (*p* < 0.05). In the convenience domain, patient satisfaction was positively correlated with junior high school (*p* = 0.006), high school/secondary/vocational school (*p* = 0.032) education, in addition, patient satisfaction was positively correlated with never smoked (*p* = 0.027), patient satisfaction was negatively correlated with CAT score (*p* < 0.0001) and home non-invasive ventilation time (*p* < 0.05). In the area of global satisfaction, patient satisfaction was positively correlated with junior high school (*p* = 0.011), in addition, patient satisfaction was negatively correlated with professional and technical personnel (*p* = 0.036), and finally patient satisfaction was negatively correlated with CAT score (*p* < 0.0001).

**Table 3:** Influencing factors of medication satisfaction of COPD patients, based on TSQM.

	Effectiveness		Side effect		Convenience		Global satisfaction	
	Coefficient	*P-*value	Coefficient	*P-*value	Coefficient	*P-*value	Coefficient	*P-*value
**Education background (Ref. primary school and below)**								
Junior high school	1.779	0.011	--	--	3.008	0.006	2.773	0.011
High school or technical or vocational school	3.262	0.001	--	--	3.353	0.032	2.890	0.064
Bachelor degree or above	2.680	0.141	--	--	−3.596	0.203	−3.358	0.235
**Profession (Ref. agriculture, forestry, animal husbandry, fishing, water production personnel)**								
A	−1.969	0.181	--	--	−4.190	0.066	−4.790	0.036
B	−1.389	0.330	--	--	−3.855	0.080	−3.573	0.105
C	−0.456	0.785	--	--	−1.824	0.480	−1.688	0.513
D	1.305	0.291	--	--	0.021	0.991	−0.239	0.901
E	5.261	0.096	--	--	3.341	0.497	2.940	0.550
F	2.062	0.003	--	--	−1.192	0.273	−1.270	0.242
**Place of residence (Ref. rural area)**								
Urban area	--	--	−1.722	0.084	--	--	--	--
**Smoking status (Ref. be smoking)**								
Previous smoking	--	--	--	--	2.041	0.114	1.502	0.243
Never smoked	--	--	--	--	3.070	0.027	2.250	0.107
CAT score	−0.357	<0.0001	−0.511	<0.0001	−0.538	<0.0001	−0.516	<0.0001
mMRC score	0.002	0.995	1.272	0.013	0.941	0.058	0.909	0.067
**Total number of acute exacerbations in the past 1 year (Ref. zero)**								
1 to 3	2.490	0.002	−0.614	0.638	--	--	--	--
≥3	−1.093	0.215	−0.883	0.565	--	--	--	--
**Total number of severe acute exacerbations in the past 1 year (Ref. zero)**								
1			1.464	0.382			1.411	0.348
≥2			−2.639	0.118			−2.890	0.053
**Home oxygen therapy time [hours/day (%)] (Ref. zero)**								
0 to 4	3.093	0.003	2.470	0.122	--	--	--	--
4 to 8	3.968	<0.0001	−2.286	0.111	--	--	--	--
>8	3.997	<0.0001	1.017	0.517	--	--	--	--
**Home non-invasive ventilation time[hours/day (%)] (Ref. zero)**								
0 to 4	−5.088	<0.0001	−5.797	0.005	−5.618	0.005	--	--
>4	−3.438	0.001	−4.609	0.005	−5.860	<0.0001	--	--

A, Professional and technical personnel; B, Production and transportation equipment operators and related personnel; C, Commercial service personnel; D, State organs, Party and mass organizations, enterprises, institutions; E, Medical and health related personnel; F, Others.

## 4. Discussion

In this multicenter cross-sectional study, we looked at medication satisfaction and its associated factors in older adult patients with chronic respiratory disease in different regions of Hunan Province, China. This focus on the older adult population is particularly relevant in the context of public health, as this demographic group often faces unique challenges related to their health and well-being. Understanding medication satisfaction among older adults is crucial for improving their overall health outcomes and ensuring effective management of chronic respiratory conditions. By investigating medication satisfaction in this specific population, we gain valuable insights into the actual utilization of medications in managing chronic respiratory disease and the level of adherence among older adult patients. Older adults often require multiple medications for various health conditions, and factors such as polypharmacy, cognitive impairment, and mobility limitations can affect their ability to adhere to prescribed medications. Recognizing and addressing these factors is essential for promoting the safe and effective use of medications among the older adult population.

In this study, the current satisfaction of older adult patients with chronic respiratory disease with the medications they use was first described, specifically demonstrated in four domains: effectiveness, side effects, convenience, and overall satisfaction. With a good treatment satisfaction score of greater than or equal to 80, only 15.9% of patients scored above 80 in the effectiveness domain, while the values in the convenience and overall satisfaction domains were 11.6% and 16.5%, respectively. The effectiveness domain included two dimensions of satisfaction—the drug’s ability to prevent treatment and the ability to relieve symptoms. This information could be used to improve the production of drugs or the selection of medications prescribed by clinicians in the future. For instance, inhalation therapy was found to be a more effective treatment option for COPD and asthma patients as it has easier access to the bronchial and alveolar systems and requires a lower dosage compared to oral or parenteral therapy (24). Inhalation therapy has the advantage of easier access to the alveolar system and lower dose compared to oral or parenteral therapy, but poor adherence due to inadequate patient inhaler technology affects disease management (25, 26), which highlights the need for further improvements in drug use by drug manufacturers. The study also revealed that a significant proportion of patients who reported side effects were dissatisfied with their ability to work physically, emotionally, and mentally with the medication used. These aspects can significantly impact the older adult population’s willingness and ability to continue their prescribed treatment regimens. Understanding these limitations and tailoring interventions to address them can help improve medication efficacy, patient adherence, and overall health outcomes in this vulnerable population. Results of a multiple linear regression analysis for overall chronic respiratory disease patients showed that education was an influential factor in patient satisfaction scores, while medication satisfaction was higher among patients in health care-related occupations. In addition, a noteworthy point is that medication satisfaction was higher among patients who had never smoked, smoking being the most prevalent risk factor for chronic respiratory disease worldwide (2), and further education on smoking cessation is needed in the future.

In addition, classification comparison showed that patients with interstitial pulmonary disease had lower satisfaction with drugs and higher reporting rate of side effects than patients with other diseases. This finding is consistent with the current situation, as there is a shortage of specific drugs available for treating interstitial lung disease. The use of “all-gold oil” hormone drugs is only effective for certain types of interstitial lung disease, and in most cases, the use of hormones is not effective and has noticeable side effects (27). However, the current specific drugs such as “Pirfenidone” and “Nintedanib” are not specific drugs in the clinical sense. Their effectiveness is only for some types of patients with interstitial lung disease, and the patients with mild to moderate disease need to be satisfied (28). Therefore, patients with interstitial lung disease face particular challenges, and the current drug options have limited effectiveness and significant side effects. The development of specific drugs for interstitial lung disease requires further research to address this critical medical need.

This study also focused on the current status and factors influencing medication satisfaction in COPD patients, where the treatment strategy is known to control inflammation at the source, stop disease progression, and treat symptoms caused by airway alveolar destruction. Therefore, COPD treatment drugs are also divided into two categories: anti-inflammatory drugs for causal treatment and bronchodilators for symptomatic treatment (29). The study conducted multiple linear regression analysis and identified several factors that influenced patients’ medication satisfaction scores. The findings indicated that patients’ education background, profession, and history of acute exacerbations were influential factors in determining medication satisfaction. The study also highlighted the role of patient disease severity, as measured by the COPD Assessment Test (CAT) score, in determining medication satisfaction. The results showed that patients with higher CAT scores were less satisfied with their medication, suggesting that current symptoms influence medication satisfaction, which could in turn affect patient adherence to treatment. The study also found that factors related to the use of home oxygen therapy was associated with medication satisfaction scores. Home oxygen therapy was found to be a good complement to patients’ medication, and it improved their medication satisfaction. These findings provide valuable insights into the factors that affect medication satisfaction in older adult patients. Healthcare providers can use this information to develop tailored interventions to improve medication satisfaction and, consequently, patient adherence to treatment.

This study has some limitations. It is an observational study, and in addition the generalization and generalization of the results may be limited because it is not a national study and there may be differences between provinces. In addition, there may be some potential selection bias as participants were limited to hospitalized patients. Cross-sectional findings cannot draw conclusions about causality, and further research is warranted to explore causality. Patient selection was based on convenience (leading to potential selection bias) and willingness to complete time-consuming patient-reported outcome (PRO) questionnaires, all of which have inherent response and assessment biases. And patients may not want to express dissatisfaction with their medication regimen to their healthcare provider, which could lead to an overestimation of satisfaction rates.

## 5. Conclusion

Multivariate analysis showed that age, education background, profession, and smoking status were independently associated with patient satisfaction. Satisfaction with medication for interstitial lung disease was lower than for other diseases at the disease-specific level. Multivariate analysis for COPD showed that education background, profession, CAT score, number of acute exacerbations and home oxygen therapy time and home non-invasive ventilation time were independently associated with patient satisfaction. Therefore, the findings of this study have the potential to provide valuable guidance for both drug manufacturers and clinicians. By reflecting the specifics of patient satisfaction with medication and considering the related influencing factors, drug manufacturers can design medications that better meet the needs and preferences of the older adult population. Clinicians, on the other hand, can utilize this information to optimize medication prescribing practices, enhance patient education and counseling, and develop strategies to improve patient adherence to medication regimens. Ultimately, such improvements can contribute to better health outcomes and quality of life for older adult individuals with chronic respiratory disease.

## Data availability statement

The raw data supporting the conclusions of this article will be made available by the authors, without undue reservation.

## Ethics statement

The studies involving human participants were reviewed and approved by Clinical Trial and Ethics Committee of the Second Xiangya Hospital of Central South University (Registration number: LYF2021012). The patients/participants provided their written informed consent to participate in this study.

## Author contributions

JW, WM, HZ, YM, and YC: conception and design. JW: interpretation of data, statistical analysis, and manuscript writing. YM and YC: revision of manuscript and administrative, technical, or material support. All authors contributed to the article and approved the submitted version.

## Funding

This work was supported by the National Natural Science Foundation of Hunan Province (no. 2022JJ30060), the Fundamental Research Funds for the Central Universities of Central South University (no. 2021zzts0369), and the Hunan Provincial Innovation Foundation for Postgraduate (CX20210371).

## Conflict of interest

The authors declare that the research was conducted in the absence of any commercial or financial relationships that could be construed as a potential conflict of interest.

## Publisher’s note

All claims expressed in this article are solely those of the authors and do not necessarily represent those of their affiliated organizations, or those of the publisher, the editors and the reviewers. Any product that may be evaluated in this article, or claim that may be made by its manufacturer, is not guaranteed or endorsed by the publisher.

## References

[1] WHO. *Chronic respiratory diseases.* (2020); Available at: https://www.who.int/health-topics/chronic-respiratory-diseases#tab=tab_1 (Accessed 22 May 2020).

[2] GBD Chronic Respiratory Disease Collaborators. Prevalence and attributable health burden of chronic respiratory diseases, 1990-2017: a systematic analysis for the global burden of disease study 2017. Lancet Respir Med. (2020) 8:585–96. doi: 10.1016/S2213-2600(20)30105-3 32526187 PMC7284317

[3] The Lancet. GBD 2017: a fragile world. Lancet. (2018) 392:1683. doi: 10.1016/S0140-6736(18)32858-7 30415747

[4] WHO. *Global Alliance against chronic respiratory disease—About GARD*. (2020); Available from: https://www.who.int/gard/en/ (Accessed 22 May 2020).

[5] Society E.R. The economic burden of lung disease*. In: European lung white book*. (2020); Available at: https://www.erswhitebook.org/chapters/the-economic-burden-of-lung-disease/ (Accessed 22 May 2020).

[6] Fong JH. Disability incidence and functional decline among older adults with major chronic diseases. BMC Geriatr. (2019) 19:323. doi: 10.1186/s12877-019-1348-z, PMID: 31752701 PMC6873710

[7] Dogra S Dunstan DW Sugiyama T Stathi A Gardiner PA Owen N. Active aging and public health: evidence, implications, and opportunities. Annu Rev Public Health. (2022) 43:439–59. doi: 10.1146/annurev-publhealth-052620-091107 34910580

[8] Jerez-Roig J Medeiros LFB Silva VAB Bezerra CLPAM Cavalcante LAR Piuvezam G. Prevalence of self-medication and associated factors in an elderly population: a systematic review. Drugs Aging. (2014) 31:883–96. doi: 10.1007/s40266-014-0217-x, PMID: 25323057

[9] Ogawa H Fujimura M Takeuchi Y Makimura K. Dealing with cough-related laryngeal sensations for a substantial reduction in chronic cough. Pulm Pharmacol Ther. (2014) 27:127–8. doi: 10.1016/j.pupt.2013.06.007, PMID: 23819969

[10] Kew KM Mavergames C Walters JA. Long-acting beta2-agonists for chronic obstructive pulmonary disease. Cochrane Database Syst Rev. (2013) 10:Cd010177. doi: 10.1002/14651858.CD010177.pub2 PMC11663505 24127118

[11] Calzetta L Ritondo BL Zappa MC Manzetti GM Perduno A Shute J. The impact of long-acting muscarinic antagonists on mucus hypersecretion and cough in chronic obstructive pulmonary disease: a systematic review. Eur Respir Rev. (2022) 31:210196. doi: 10.1183/16000617.0196-2021, PMID: 35508331 PMC9488979

[12] Boardman C Chachi L Gavrila A Keenan CR Perry MM Xia YC. Mechanisms of glucocorticoid action and insensitivity in airways disease. Pulm Pharmacol Ther. (2014) 29:129–43. doi: 10.1016/j.pupt.2014.08.008, PMID: 25218650

[13] Maselli DJ Keyt H Restrepo MI. Inhaled antibiotic therapy in chronic respiratory diseases. Int J Mol Sci. (2017) 18:1062. doi: 10.3390/ijms18051062, PMID: 28509852 PMC5454974

[14] Global Initiative for Chronic Obstruction Lung Disease. Global strategy for the diagnosis, management and prevention of chronic obstructive pulmonary disease 2023 report. Available at: https://goldcopd.org/2023-gold-report/ (Accessed 15 November 2022).

[15] Blaschke TF Osterberg L Vrijens B Urquhart J. Adherence to medications: insights arising from studies on the unreliable link between prescribed and actual drug dosing histories. Annu Rev Pharmacol Toxicol. (2012) 52:275–301. doi: 10.1146/annurev-pharmtox-011711-113247, PMID: 21942628

[16] Contoli M Rogliani P Di Marco F Braido F Corsico AG Amici CA. Satisfaction with chronic obstructive pulmonary disease treatment: results from a multicenter, observational study. Ther Adv Respir Dis. (2019) 13:1753466619888128. doi: 10.1177/1753466619888128 31760881 PMC6878607

[17] Atkinson MJ Kumar R Cappelleri JC Hass SL. Hierarchical construct validity of the treatment satisfaction questionnaire for medication (TSQM version II) among outpatient pharmacy consumers. Value Health. (2005) 8:S9–S24. doi: 10.1111/j.1524-4733.2005.00066.x, PMID: 16336491

[18] Atkinson MJ Sinha A Hass SL Colman SS Kumar RN Brod M. Validation of a general measure of treatment satisfaction, the treatment satisfaction questionnaire for medication (TSQM), using a national panel study of chronic disease.Pdf. Health Qual Life Outcomes. (2004) 2:12. doi: 10.1186/1477-7525-2-12, PMID: 14987333 PMC398419

[19] Lee SH Kim YG Lee SG Lee SH Kim YJ Jeon JY. Treatment pattern, satisfaction, and productivity loss of patients with ankylosing spondylitis treated with tumor necrosis factor inhibitors in Korea: a multicenter cross-sectional observational study. Int J Rheum Dis. (2022) 25:523–31. doi: 10.1111/1756-185X.14304, PMID: 35187866 PMC9303183

[20] Osmanovic A Ranxha G Kumpe M Wurster CD Stolte B Cordts I. Treatment satisfaction in 5q-spinal muscular atrophy under nusinersen therapy. Ther Adv Neurol Disord. (2021) 14:1756286421998902. doi: 10.1177/1756286421998902 33747131 PMC7940734

[21] Kawahito Y Takakubo Y Morinobu A Matsubara N Nagy O Sugiyama E. Patient satisfaction, preferences, expectations, characteristics, and impact of suboptimal control of rheumatoid arthritis: a subgroup analysis of Japanese patients from a large international cohort study (SENSE). PLoS One. (2021) 16:e0259389. doi: 10.1371/journal.pone.0259389, PMID: 34780502 PMC8592402

[22] Yabin S Hongxia L Lixin Y Yun M Guirong Y Sisi W. Transcultural adaptation of the treatment satisfaction questionnaire for medication. Chin Nurs Manag. (2018) 18:612–6. doi: 10.3969/j.issn.1672-1756.2018.05.00823

[23] Yin R Cao H Fu T Zhang Q Zhang L Li L. The rate of adherence to urate-lowering therapy and associated factors in Chinese gout patients: a cross-sectional study. Rheumatol Int. (2017) 37:1187–94. doi: 10.1007/s00296-017-3746-x, PMID: 28551724

[24] Gregoriano C Dieterle T Breitenstein AL Dürr S Baum A Maier S. Use and inhalation technique of inhaled medication in patients with asthma and COPD: data from a randomized controlled trial. Respir Res. (2018) 19:237. doi: 10.1186/s12931-018-0936-3, PMID: 30509268 PMC6276152

[25] Melani AS Bonavia M Cilenti V Cinti C Lodi M Martucci P. Inhaler mishandling remains common in real life and is associated with reduced disease control. Respir Med. (2011) 105:930–8. doi: 10.1016/j.rmed.2011.01.005, PMID: 21367593

[26] Darbà J Ramírez G Sicras A García-Bujalance L Torvinen S Sánchez-de la Rosa R. Identification of factors involved in medication compliance: incorrect inhaler technique of asthma treatment leads to poor compliance. Patient Prefer Adherence. (2016) 10:135–45. doi: 10.2147/PPA.S95303, PMID: 26929605 PMC4754100

[27] Raghu G Remy-Jardin M Richeldi L Thomson CC Inoue Y Johkoh T. Idiopathic pulmonary fibrosis (an update) and progressive pulmonary fibrosis in adults: an official ATS/ERS/JRS/ALAT clinical practice guideline. Am J Respir Crit Care Med. (2022) 205:e18–47. doi: 10.1164/rccm.202202-0399ST, PMID: 35486072 PMC9851481

[28] Finnerty JP Ponnuswamy A Dutta P Abdelaziz A Kamil H. Efficacy of antifibrotic drugs, nintedanib and pirfenidone, in treatment of progressive pulmonary fibrosis in both idiopathic pulmonary fibrosis (IPF) and non-IPF: a systematic review and meta-analysis. BMC Pulm Med. (2021) 21:411. doi: 10.1186/s12890-021-01783-1, PMID: 34895203 PMC8666028

[29] Singh D. Pharmacological treatment of stable chronic obstructive pulmonary disease. Respirology. (2021) 26:643–51. doi: 10.1111/resp.14046 33829619

