# Supplementary material for: Satisfaction with medication in older adult patients with chronic respiratory diseases: a multicenter cross-sectional observational study
Source: Front Public Health. 2023 Aug 21;11:1168249. doi: 10.3389/fpubh.2023.1168249 (PMC10476521; doi:10.3389/fpubh.2023.1168249)
Supplement: Supplementary file 1 [file Table_1.DOCX]

**Supplementary Table 1**. Characteristics of the COPD Patients

| **Characteristics** | **Total(N=2218)** |
| --- | --- |
| Age (years), mean (SD) | 70.94±9.68 |
| Sex, n (%) |  |
| Male | 1722(78.1) |
| Female | 496(21.9) |
| Education background, n (%) |  |
| primary school and below | 1145(53.6) |
| Junior high school | 702(31.4) |
| High school or technical or vocational school | 296(12.0) |
| Bachelor degree or above | 75(3.0) |
| Local Area, n (%) |  |
| Developed area | 1617(70.2) |
| Less developed area | 601(29.8) |
| Profession, n(%) |  |
| Agriculture, forestry, animal husbandry, fishing, water production personnel | 1006(47.2) |
| Professional and technical personnel | 112(4.5) |
| Production and transportation equipment operators and related personnel | 113(5.2) |
| Commercial service personnel; | 80(3.4) |
| State organs, Party and mass organizations, enterprises, institutions; | 187(7.7) |
| Medical and health related personnel; | 21(0.9) |
| Others | 699(31.1) |
| Place of residence, n(%) |  |
| Urban area | 754(31.9) |
| Rural area | 1464(68.2) |
| Smoking status, n(%) |  |
| Be smoking | 403(19.2) |
| Previous smoking | 1136(50.3) |
| Never smoked | 679(30.5) |
| CAT score (xˉ±s) | 14.58±7.45 |
| mMRC score (xˉ±s) | 1.71±1.16 |
| Total number of acute exacerbations in the past 1 year, n(%) |  |
| 0 | 260(12.9) |
| 1 to 3 | 1452(66.5) |
| ≥3 | 506(20.6 |
| Total number of severe acute exacerbations in the past 1 year, n(%) |  |
| 0 | 299(13.5) |
| 1 | 775(34.9) |
| ≥2 | 1144(51.6) |
| Home oxygen therapy, n(%) | 902(37.3) |
| Home oxygen therapy time [hours/day (%)] |  |
| 0 | 1316(59.3) |
| 0 to 4 | 243(11.0) |
| 4 to 8 | 348(15.7) |
| >8 | 311(14.0) |
| Home use of ventilator, n(%) | 357(14.9) |
| Time spent on ventilator at home [hours/day (%)] |  |
| 0 | 1861(83.9) |
| 0 to 4 | 127(5.7) |
| 4 to 8 | 152(6.9) |
| >8 | 78(3.5) |

**Supplementary Table 2**. The current status of TSQM II application in the world.

| References | Year | Country | Research purpose | Research method |
| --- | --- | --- | --- | --- |
| Jiang et al [1]. | 2020 | China | Investigate physician and rheumatoid arthritis patient satisfaction with treatment and explore potential factors. | Cross-sectional study of 12 centers nationwide, using TSQM II. |
| Watanabe-Fujinuma, Emi et al [2]. | 2019 | Japan | Assessment of psychometric properties of Japanese patients with atrial fibrillation. | In a post-marketing surveillance study of the direct oral anticoagulant rivaroxaban in Japan, anticoagulant block therapy scale and TSQM-II data were collected from 534 patients with atrial fibrillation. |
| Oosterhaven, Jart A F et al [3]. | 2022 | Netherlands | Measuring perceived eczema control and treatment satisfaction in patients with atopic dermatitis treated with dupilumab for 16 - 52 weeks. | Collection of atopic dermatitis control tests, overview of atopic eczema (Recap), TSQM II, and reported outcome measures in patients with atopic dermatitis. |
| Salmasi, Shahrzad et al [4]. | 2021 | Canada | To assess the satisfaction of patients with atrial fibrillation with oral anticoagulant use over time and to identify relevant patient characteristics. | Satisfaction was measured using the TSQM II and the Anticoagulant Therapy Scale (ACTS) for eligible patients. |
| Fallu, Angelo et al [5]. | 2016 | Canada | To evaluate adult attention-deficit/hyperactivity disorder subject reported outcomes when switched from a stable dose of CONCERTA(®) to the same dose of generic Novo-methylphenidate ER-C(®). | Primary efficacy was assessed through the use of the TSQM-II. |
| Gupta, Shaloo et al [6]. | 2015 | USA | The authors sought to characterize health-related quality of life, medication adherence, productivity losses, and treatment satisfaction associated with modifications to opioid therapy due to opioid-induced constipation (OIC). | Patients were assessed for constipation quality of life and symptoms, Morisky medication adherence Scale, work efficiency and activity disorders, and TSQM II for OIC treatment. |
| Guglieri, Michela et al [7]. | 2022 | Germany, USA, Italy, UK, Canada | To compare efficacy and adverse effects of the 3 most frequently prescribed corticosteroid regimens in boys with Duchenne muscular dystrophy. | Overall participant or parent satisfaction with treatment was assessed by TSQM II. |
| Markman, John D et al [8]. | 2022 | USA | To focus on the response of patients with chronic low back pain to tanezumab and tramadol treatment by assessing secondary measures of pain, interference with daily function, overall disease status, and treatment satisfaction. | TSQM II was assessed at week 16 and week 56. |

1. Jiang, N., et al., *Satisfaction of Patients and Physicians with Treatments for Rheumatoid Arthritis: A Population-Based Survey in China.* Patient Prefer Adherence, 2020. **14**: p. 1037-1047.

2. Watanabe-Fujinuma, E., et al., *Psychometric validation of anti-clot treatment scale and treatment satisfaction questionnaire for medication version II in Japanese patients with atrial fibrillation.* J Med Econ, 2019. **22**(8): p. 798-805.

3. Oosterhaven, J.A.F., et al., *Eczema control and treatment satisfaction in atopic dermatitis patients treated with dupilumab - a cross-sectional study from the BioDay registry.* J Dermatolog Treat, 2022. **33**(4): p. 1986-1989.

4. Salmasi, S., et al., *Satisfaction With Oral Anticoagulants Among Patients With Atrial Fibrillation: A Prospective Observational Study.* CJC Open, 2021. **3**(11): p. 1347-1356.

5. Fallu, A., et al., *A randomized, double-blind, cross-over, phase IV trial of oros-methylphenidate (CONCERTA(®)) and generic novo-methylphenidate ER-C (NOVO-generic).* Ther Adv Psychopharmacol, 2016. **6**(4): p. 237-51.

6. Gupta, S., et al., *Impact of constipation on opioid therapy management among long-term opioid users, based on a patient survey.* J Opioid Manag, 2015. **11**(4): p. 325-38.

7. Guglieri, M., et al., *Effect of Different Corticosteroid Dosing Regimens on Clinical Outcomes in Boys With Duchenne Muscular Dystrophy: A Randomized Clinical Trial.* Jama, 2022. **327**(15): p. 1456-1468.

8. Markman, J.D., et al., *Clinical Meaningfulness of Response to Tanezumab in Patients with Chronic Low Back Pain: Analysis From a 56-Week, Randomized, Placebo- and Tramadol-Controlled, Phase 3 Trial.* Pain Ther, 2022. **11**(4): p. 1267-1285
